# Signal Detection Theory as a Novel Tool to Understand Cognitive Fatigue in Individuals With Multiple Sclerosis

**DOI:** 10.3389/fnbeh.2022.828566

**Published:** 2022-03-16

**Authors:** Cristina A. F. Román, John DeLuca, Bing Yao, Helen M. Genova, Glenn R. Wylie

**Affiliations:** ^1^Rocco Ortenzio Neuroimaging Center, Kessler Foundation, West Orange, NJ, United States; ^2^Department of Physical Medicine and Rehabilitation, Rutgers University, Newark, NJ, United States; ^3^Department of Neurology, New Jersey Medical School, Newark, NJ, United States; ^4^Department of Veterans Affairs, The War Related Illness and Injury Center, New Jersey Healthcare System, East Orange, NJ, United States

**Keywords:** multiple sclerosis, cognitive fatigue, signal detection theory, neuroimaging, striatum

## Abstract

Multiple Sclerosis (MS) affects 2.8 million persons worldwide. One of the most persistent, pervasive, and debilitating symptoms of MS is cognitive fatigue. While this has been known for over a century, cognitive fatigue has been difficult to study because patients’ subjective (self-reported) cognitive fatigue has consistently failed to correlate with more objective measures, such as reaction time (RT) and accuracy. Here, we investigated whether more nuanced metrics of performance, specifically the metrics of Signal Detection Theory (SDT), would show a relationship to cognitive fatigue even if RT and accuracy did not. We also measured brain activation to see whether SDT metrics were related to activation in brain areas that have been shown to be sensitive to cognitive fatigue. Fifty participants (30 MS, 20 controls) took part in this study and cognitive fatigue was induced using four blocks of a demanding working memory paradigm. Participants reported their fatigue before and after each block, and their performance was used to calculate SDT metrics (Perceptual Certainty and Criterion) and RT and accuracy. The results showed that the SDT metric of Criterion (i.e., response bias) was positively correlated with subjective cognitive fatigue. Moreover, the activation in brain areas previously shown to be related to cognitive fatigue, such as the striatum, was also related to Criterion. These results suggest that the metrics of SDT may represent a novel tool with which to study cognitive fatigue in MS and other neurological populations. These results hold promise for characterizing cognitive fatigue in MS and developing effective interventions in the future.

## Introduction

Multiple sclerosis (MS) is a degenerative autoimmune disease that impacts approximately 2.8 million people worldwide and one-million people in the United States (Wallin et al., 2019; Walton et al., 2020). Fatigue, including cognitive fatigue, is one of the most common and debilitating symptoms reported by persons with MS (pwMS; Freal et al., 1984; Fisk et al., 1994), impacting employment, quality of life, psychological state, and daily functioning (Nagaraj et al., 2013; Coyne et al., 2015; Tabrizi and Radfar, 2015; Gullo et al., 2019; Rooney et al., 2019). While there is no universally accepted definition of cognitive fatigue, Chaudhuri and Behan (2000) define cognitive fatigue, or “central fatigue,” as an “enhanced perception of effort and limited endurance of sustained physical and mental activities…[it is a] difficulty in initiation of or sustaining voluntary activities” (pp. 978). Cognitive fatigue can be classified as “state” fatigue (i.e., fatigue ratings in the moment, often measured during or soon after a cognitive demanding task) or “trait” fatigue (i.e., retrospective fatigue ratings, often as reported on self-report inventories) and further broken down into primary and secondary fatigue (Johnson, 2008; Kos et al., 2008).

Quantifying cognitive fatigue is a challenge. To-date, most studies examining cognitive fatigue in pwMS have relied on subjective, self-report inventories, which have several limitations (e.g., sensitive to recall and response bias, poor sensitivity to change over time, not specific to cognitive fatigue). In addition, fatigue scores derived from self-report inventories do not often correlate with performance-based measures thought to provide a more objective metric of cognitive fatigue, such as reaction time and accuracy (Torres-Harding and Jason, 2005; Claros-Salinas et al., 2013; Kluger et al., 2013). This incongruence has created barriers to characterizing cognitive fatigue, identifying its neural signatures, and developing clinical interventions for pwMS.

Signal Detection Theory (SDT) holds promise as an objective measure of cognitive fatigue that may also align with the subjective experiences of pwMS. SDT proposes that one’s ability to detect a stimulus is not only based on the intensity of the stimulus itself but also the psychological or physiological state of the person observing the stimulus. Fatigue has been linked to decrements in variables derived from SDT, namely Perceptual Certainty (*d’*) and Criterion (beta) (Green and Swets, 1966; Lynn and Barrett, 2014; Wylie et al., 2020). Perceptual Certainty refers to the ability to discriminate targets from non-targets, while Criterion is related to the amount of evidence needed to determine whether a stimulus is a target or not. An individual will set their Criterion based on the payoff matrix: in situations where there is a large incentive to respond to target stimuli and little cost for false alarms, subjects will adopt a liberal Criterion and respond to anything that might be a target stimulus. Conversely, when false alarms are discouraged and correct responses are only modestly encouraged, subjects will adopt a more conservative Criterion and respond only when they are confident a stimulus is a target (Green and Swets, 1966). In 2002, Matthews and Desmond found an inverse relationship between Perceptual Certainty and fatigue during a simulated “difficult” driving task (relative to an easier task), such that Perceptual Certainty decreased as fatigue increased (in a non-neurological sample). A similar pattern was observed during a more recent study from our group in which cognitive fatigue was induced by repetitive performance of a working memory task while simultaneous functional magnetic resonance imaging (fMRI) data were collected in a group of volunteers without neurological or psychiatric histories (Wylie et al., 2020). Cognitive “state” fatigue was evaluated throughout the task and mean values were calculated from blocks in which at least some fatigue was reported. As expected, self-reported cognitive fatigue did not correlate with basic performance variables, such as reaction time or accuracy, but it did correlate with Perceptual Certainty and Criterion. These results not only support the use of SDT metrics to examine cognitive fatigue, but they are an integral first step to understanding the underlying link between subjective state and objective fatigue using a novel theoretical approach.

Several connections between subjective state and objective cognitive fatigue have been proposed over the last few decades, with changes in the payoff matrix between effort and reward serving as a viable explanation. The idea that fatigue is linked to changes in the payoff matrix stems from studies showing that performance and cognitive fatigue can be improved through increased reward/motivation. Lorist et al. (2009), for example, induced cognitive fatigue during a demanding two-hour long task. Results showed an inverse relationship between fatigue ratings and performance, such that as fatigue increased, performance decreased (i.e., more errors, slower reaction time). When participants were offered a monetary reward to improve their performance after two-hours, however, their performance improved substantially, suggesting they were motivated to *overcome* their fatigue in light of increased reward. Evidence of fatigue stemming from changes in the payoff matrix have also been demonstrated by several other groups (see Dobryakova et al., 2013; Dobryakova et al., 2015; Wylie et al., 2017b; Müller and Apps, 2019). Interestingly, the introduction of greater reward does not just influence performance, but it reduces reported fatigue as well (Matthews and Desmond, 2002; Boksem et al., 2006; Lorist et al., 2009). SDT predicts that changes in the payoff matrix are related to changes in Criterion.

Identifying neural signatures of the relationship between objective and state fatigue is also crucial. Chaudhuri and Behan (2000) were among the first to propose that cognitive fatigue stems from malfunctions of non-motor processes of the basal ganglia. Subsequent neuroimaging research followed suit, showing functional and structural evidence of the association between cognitive fatigue and abnormalities involving an underlying cortico-striato-thalamo-cortical loop (Chalah et al., 2015; Ayache and Chalah, 2017), namely the largest structure of the basal ganglia called the striatum (Chaudhuri and Behan, 2004; DeLuca et al., 2008; Dobryakova et al., 2013, 2017; Nakagawa et al., 2016), with more recent studies linking the basal ganglia to SDT metrics (Wylie et al., 2020). More specifically, the caudate, which is in the dorsal striatum and involved in both motor (i.e., planning and executing voluntary movement) and non-motor processes (i.e., learning and memory, reward, motivation, and emotion; Driscoll et al., 2020), appears to be an integral player in cognitive fatigue across neurological populations. Stroke-induced caudate lesions, for example, have been shown to be independently associated with poststroke fatigue (Tang et al., 2013). In addition, Wylie et al. (2017a) compared a group of persons with moderate to severe traumatic brain injury (TBI) to controls and found that the TBI group not only reported more fatigue, but they evidenced a positive correlation between fatigue and activation in the caudate tail when partaking in a “difficult” working memory task (compared to a control task). Subsequent confirmatory analyses on a separate dataset showed the same pattern for persons with TBI during a processing speed task. The importance of the caudate has also been evidenced in work with pwMS. Andreasen et al. (2010) found fatigued pwMS to have greater atrophy in areas related to attentional control, including the head of the caudate. Further, Akbar et al. (2020) demonstrated that greater increases in functional connectivity between the caudate and left parietal region following progressive resistance exercise training in pwMS correlated with greater decreases in cognitive fatigue. Thus, the striatum, particularly the caudate, has emerged as a potential neural substrate of both subjective and objective cognitive fatigue, though their relationships with SDT metrics in pwMS remains largely unknown.

The current study examined a group of pwMS and controls (i.e., no neurological history) to achieve the following aims: (1) investigate whether SDT metrics (i.e., Perceptual Certainty, Criterion) are better measures of cognitive fatigue than basic performance metrics (i.e., reaction time, accuracy); (2) determine the relationship between subjective state and objective (SDT metrics) cognitive fatigue; and (3) identify structural and functional neural substrates that are sensitive to cognitive state fatigue and SDT metrics. Given previous work, we hypothesize that SDT metrics, namely Criterion, will be associated with subjective state cognitive fatigue due to changes in the payoff matrix, while basic performance metrics will not. In addition, we expect that the basal ganglia, namely the caudate, will play an integral role in this relationship.

## Materials and Methods

### Participants

This study included 50 participants, 30 of whom had clinically definite MS according to McDonald criteria (Polman et al., 2005) and 20 controls with no neurological history who were matched on age and education (see Table 1). The groups were not matched on sex (see Table 1), so sex was therefore included in all group-level analyses as a covariate. All study procedures were conducted in English and approved by Kessler Foundation’s Institutional Review Board.

**TABLE 1:** Demographic table.

	MS (*n* = 30)	Control (*n* = 20)	Statistic	*p*-value
Age (years)	47.4 (10.6)	46.9 (10.9)	*t*(57.6) = −0.20	*p* > 0.8
Education (years)	16.0 (2.2)	15.5 (2.4)	*t*(57.7) = –0.83	*p* > 0.4
Sex (women/men)	27/3	15/5	**χ**^2^ (1) = 9.60	*p* < 0.01
Duration of illness (months)	135.1 (72.0)	n/a		
Disease course				
Relapsing- remitting	24	n/a		
Primary- progressive	1	n/a		
Secondary- progressive	3	n/a		
Progressive- relapsing	2	n/a		
In-scanner motion				
Rotation (degrees)	0.01	0.02	*F*(1,39.5) = 0.12	*p* = 0.73
Translation (mm)	0.80	0.82	*F*(1,38.3) = 0.02	*p* = 0.88
VAS-F × Rating				
Baseline	30.2	13.5		
Post-block1	31.6	9.9		
Post-block2	36.8	7.9		
Post-block3	38.6	11.7		
Post-block4	42.8	15.1		

*For the groups, the means are given with the standard deviation in parentheses.*

### Neuroimaging Acquisition

Neuroimaging data collection began on a 3-Tesla Siemens Allegra scanner (30 MS participants; 18 control participants) and was completed on a 3-Tesla Siemens Skyra scanner (2 control participants). For this reason, a regressor for scanner was included in all group-level analyses, as has been done in previous research utilizing more than one scanner (Stonnington et al., 2008; Biswal et al., 2010; Wylie et al., 2019). A T2*-weighted Echo Planar sequence was used to collect functional images during four blocks, with 140 brain volume acquisitions per block (Allegra: echo time = 30 ms; repetition time = 2000 ms; field of view = 22 cm; flip angle = 80°; slice thickness = 4 mm, 32 slices, matrix = 64 × 64, in-plane resolution = 3.438 × 3.438 mm^2^; Skyra: echo time = 30 ms; repetition time = 2000 ms; field of view = 22 cm; flip angle = 90°; slice thickness = 4 mm, 32 slices, matrix = 92 × 92, in-plane resolution = 2.391 × 2.391 mm^2^). A high-resolution magnetization prepared rapid gradient echo (MPRAGE) image was also acquired (Allegra: TE = 4.38 ms; TR = 2000 ms, FOV = 220 mm; flip angle = 8°; slice thickness = 1 mm, NEX = 1, matrix = 256 × 256, in-plane resolution = 0.859 × 0.859 mm^2^; Skyra: TE = 3.43 ms; TR = 2100 ms, FOV = 256 mm; flip angle = 9°; slice thickness = 1 mm, NEX = 1, matrix = 256 × 256, in-plane resolution = 1 × 1 mm^2^) and was used to register the functional data into standard MNI space for group analysis and for the volumetric analyses.

### Behavioral Paradigm and Data

Behavioral data acquisition and stimulus presentation was administered using the E-Prime software (Schneider et al., 2002). During the fMRI scan, participants were presented with the 2-back condition of the n-back working memory task. Though vigilance/sustained attention tasks are often used in investigations of cognitive fatigue, we chose a demanding working memory task (i.e., n-back) to increase the cognitive load and induce greater cognitive fatigue in our participants. In addition, we wanted to capture any decrements in performance which are more likely to occur when greater cognitive effort (i.e., working memory) is required (Parasuraman, 1979; Baddeley et al., 1999). For our working memory task, four blocks of the 2-back task were collected, with 65 trials per block. During the 2-back task, letters were presented on the screen, one at a time, and participants were asked to respond every time a presented letter was the same as the letter presented two trials before (e.g., R N Q N…). Letters were presented in white (Arial 72-point font) on a black background. Of the 26 letters in the English alphabet, the following were used with equal frequency: A B C D F H J K M N P Q R S T V Z. The other letters were excluded to enhance the discriminability of the letters used. The letter stimuli remained on the screen for 1.5 s, followed by a 500 ms inter-trial interval (ITI), and the time between successive trials was jittered to allow for the data to be deconvolved as an event related design. The jittering was optimized using the Optseq2 program^1^ and was achieved by inserting between zero and six null events between successive trials. The duration of each null event was a multiple of the length of the trial (2 s), drawn from a distribution following a power function. Most ITIs were 500 ms (zero null events), followed by 2 s (one null event) and so on. The average ITI was 1587.87 ms (± 1769.7). All participants practiced the task prior to the scanning session.

To ensure comparable stimulation across participants, the stimuli always remained on the screen for 1.5 s (that is, the stimuli were not removed when participants responded), and each run lasted the same amount of time (260 s). The average amount of time between successive blocks was 2 min. 04 s, (S.D. = 2 min. 17 s).

The following behavioral data were analyzed: overall accuracy, which was the number of trials in which the correct response was made divided by the total number of trials, the reaction times (RTs) of the correct trials, and signal detection metrics. Signal detection analysis was used to separate discrimination certainty from response bias – factors that can independently affect accuracy (Macmillan and Creelman, 2004; Anderson et al., 2011). The ability to correctly identify target stimuli was measured using the discriminability index (d’), calculated as (*z*FA – *z*HR], where *z* is the inverse of the standard normal cumulative distribution, FA is the false-alarm rate (the proportion of responses made to stimuli that were not targets), and HR is the hit rate (the proportion of correct identifications of target stimuli). In the context of this experiment, where all stimuli were readily discernable, d’ is best thought of as Perceptual Certainty rather than as sensitivity to stimulation. Response bias was measured using Criterion, calculated as −1/2[*z*HR + *z*FA] with higher values (fewer false alarms and fewer hits) indicating reduced response bias, or more conservative responding. Lower Criterion values (more hits and more false alarms) indicated increased response bias and more liberal responding.

### Visual Analogue Scale-F

To evaluate the level of on-task or “state” fatigue, participants were presented with a visual analogue scale (VAS) before and after each block of the 2-back task. Participants were asked: “How mentally fatigued are you right now?” and were asked to indicate their level of fatigue on a scale from 0 to 100, with 0 being not at all fatigued and 100 being extremely fatigued. To mask the purpose of the study, five additional VASs were administered as well, in randomized order. These assessed happiness, sadness, pain, tension, and anger.

Because VAS-F scores were obtained before and after each block, the amount of fatigue during each block was estimated by using the mean of the scores before and after the relevant block; this value was used in the correlational analyses. Furthermore, because the VAS-F scores were skewed, they were transformed using the Box-Cox method to ensure that assumptions of normality were not violated (Box and Cox, 1964). The Box-Cox method is a power transformation in which a range of power transformations are considered and the one that best transforms the data into a normal distribution is selected.

## Analyses

Prior to analysis, each variable was assessed for normality both by visual inspection and using the Agostino test (D’Agostino, 1970). In those cases when the data were found to be skewed, they were transformed using a Box-Cox transformation.

### Visual Analogue Scale-F

For the analysis of the VAS-F scores, a Linear Mixed Effects analysis [LME; using the R statistical package (version 3.4.3)] was used. Group (MS vs. control) was a between-participants factor, the within-participants factor was Rating (ratings 1–5), and Sex was included as a covariate (fixed effect); participants was included as a random factor.

### RT and Accuracy

Reaction time and accuracy were analyzed with an LME that included the factor of Group (MS vs. control); the VAS-F scores were included as a quantitative variable and Sex (female vs. male) and Run (runs 1–4) were included as fixed effects; participant was included as a random factor.

### Signal Detection Theory Measures (Perceptual Certainty and Criterion)

For each of the SDT measures [certainty (d’) and response bias (Criterion)], an LME was used with the factor of Group (MS vs. control), VAS-F (as a were quantitative variable); Sex (female vs. male) and Run (runs 1–4) were included as fixed effects; participant was included as a random factor.

### Neuroimaging

Results included in this manuscript come from preprocessing performed using *fMRIPrep* 1.4.1 (Esteban et al., 2019; RRID:SCR_016216), which is based on *Nipype* 1.2.0 (Gorgolewski et al., 2011; RRID:SCR_002502).

For anatomical preprocessing, the T1-weighted (T1w) image from each participant was corrected for intensity non-uniformity (INU) with N4BiasFieldCorrection (Tustison et al., 2010) distributed with ANTs 2.2.0 (Avants et al., 2008; RRID:SCR_004757), and used as T1w-reference throughout the workflow. The T1w-reference was then skull-stripped with a *Nipype* implementation of the antsBrainExtraction.sh workflow (from ANTs), using OASIS30ANTs as target template. Brain tissue segmentation of cerebrospinal fluid (CSF), white-matter (WM) and gray-matter (GM) was performed on the brain-extracted T1w using FAST (FSL 5.0.9, RRID:SCR_002823; Jenkinson et al., 2012). Volume-based spatial normalization to one standard space (MNI152NLin2009cAsym) was performed through non-linear registration with antsRegistration (ANTs 2.2.0), using brain-extracted versions of both T1w reference and the T1w template. The following template was selected for spatial normalization: *ICBM 152 Non-linear Asymmetrical template version 2009c* (Fonov et al., 2011; RRID:SCR_008796; TemplateFlow ID: MNI152NLin2009cAsym).

For functional data preprocessing each of the eight BOLD runs found per participant (across all tasks and sessions), the following preprocessing was performed. First, a reference volume and its skull-stripped version were generated using a custom methodology of *fMRIPrep*. The BOLD reference was then co-registered to the T1w reference using flirt (FSL 5.0.9; Jenkinson et al., 2002) with the boundary-based registration (Greve and Fischl, 2009) cost-function. Co-registration was configured with nine degrees of freedom to account for distortions remaining in the BOLD reference. Head-motion parameters with respect to the BOLD reference (transformation matrices, and six corresponding rotation and translation parameters) are estimated before any spatiotemporal filtering using mcflirt (FSL 5.0.9; Jenkinson et al., 2002). BOLD runs were slice-time corrected using 3dTshift from AFNI 20160207 (Cox and Hyde, 1997; RRID:SCR_005927). The BOLD time-series (including slice-timing correction when applied) were resampled onto their original, native space by applying a single, composite transform to correct for head-motion and susceptibility distortions. These resampled BOLD time-series will be referred to as *preprocessed BOLD in original space*, or just *preprocessed BOLD*. The BOLD time-series were resampled into standard space, generating a *preprocessed BOLD run in (“MNI152NLin2009cAsym”) space*. First, a reference volume and its skull-stripped version were generated using a custom methodology of *fMRIPrep*. Several confounding time-series were calculated based on the *preprocessed BOLD*: framewise displacement (FD), DVARS and three region-wise global signals. FD and DVARS are calculated for each functional run, both using their implementations in *Nipype* (following the definitions by Power et al., 2014). The three global signals are extracted within the CSF, the WM, and the whole-brain masks. Additionally, a set of physiological regressors were extracted to allow for component-based noise correction (*CompCor*; Behzadi et al., 2007). Principal components are estimated after high-pass filtering the *preprocessed BOLD* time-series (using a discrete cosine filter with 128 s cut-off) for the two *CompCor* variants: temporal (tCompCor) and anatomical (aCompCor). tCompCor components are then calculated from the top 5% variable voxels within a mask covering the subcortical regions. This subcortical mask is obtained by heavily eroding the brain mask, which ensures it does not include cortical GM regions. For aCompCor, components are calculated within the intersection of the aforementioned mask and the union of CSF and WM masks calculated in T1w space, after their projection to the native space of each functional run (using the inverse BOLD-to-T1w transformation). Components are also calculated separately within the WM and CSF masks. For each CompCor decomposition, the *k* components with the largest singular values are retained, such that the retained components’ time series are sufficient to explain 50 percent of variance across the nuisance mask (CSF, WM, combined, or temporal). The remaining components are dropped from consideration. The head-motion estimates calculated in the correction step were also placed within the corresponding confounds file. The confound time series derived from head motion estimates and global signals were expanded with the inclusion of temporal derivatives and quadratic terms for each (Satterthwaite et al., 2013). Frames that exceeded a threshold of 0.5 mm FD or 1.5 standardized DVARS were annotated as motion outliers. All resamplings can be performed with *a single interpolation step* by composing all the pertinent transformations (i.e., head-motion transform matrices, susceptibility distortion correction when available, and co-registrations to anatomical and output spaces). Gridded (volumetric) resamplings were performed using antsApplyTransforms (ANTs), configured with Lanczos interpolation to minimize the smoothing effects of other kernels (Lanczos, 1964). Non-gridded (surface) resamplings were performed using mri_vol2surf (FreeSurfer).

The resulting data were then deconvolved. In the deconvolution, signal drift was modeled with a set of basis functions; the motion parameters were used as regressors of no interest; and TRs with motion exceeding 0.5 mm were excluded from analysis. The regressors of interest were the correct trials of each block. Each block was deconvolved separately, and the coefficient of fit of the correct trials were entered into the group-level analysis.

Because correlations were found between d’ and VAS-F, Criterion and VAS-F, and between d’ and Criterion three group-level analyses were conducted: one for VAS-F, one for d’ and one for Criterion. In all cases, an LME was used (3dLME from the AFNI suite of processing tools) with the factors of Task (0-back vs. 2-back), and Run (runs 1–4 of each task), and with participant included as a random factor. For the analysis of VAS-F, the VAS-F scores were included as a quantitative variable (using the same transformed and averaged values as were used for the SDT analyses). For the analysis of d’, the d’ scores were included as a quantitative variable. For the analysis of Criterion, the Criterion scores were included as a quantitative variable. To see whether brain areas that were sensitive to fatigue were also sensitive to d’ and to Criterion, two additional analyses were conducted: one assessed the overlap of regions sensitive to VAS-F and d’, and the other assessed the overlap between regions sensitive to VAS-F and Criterion.

The results of these analyses were corrected for multiple comparisons by using an individual voxel probability threshold of *p* < 0.005 and a cluster threshold of 14 voxels (voxel dimension = 3.4 × 3.4 × 4 mm). Monte Carlo simulations, using 3dClustSim (version AFNI_17.2.16, compile date: Sept 19, 2017) showed this combination to result in a corrected alpha level of *p* < 0.05.

## Results

### Analysis of Movement

Because it has been shown that movement artifact can be larger in clinical samples relative to controls (Wylie et al., 2014), we extracted the parameters of maximum movement from each run (the translation and rotation) for analysis. The analysis was an LME with the factors of Group (MS vs. control) and Run (run 1–4), with Sex included as a covariate. As Table 1 shows, there was no significant difference in movement between the groups.

### Analysis of Visual Analogue Scale-F

**FIGURE 1:**
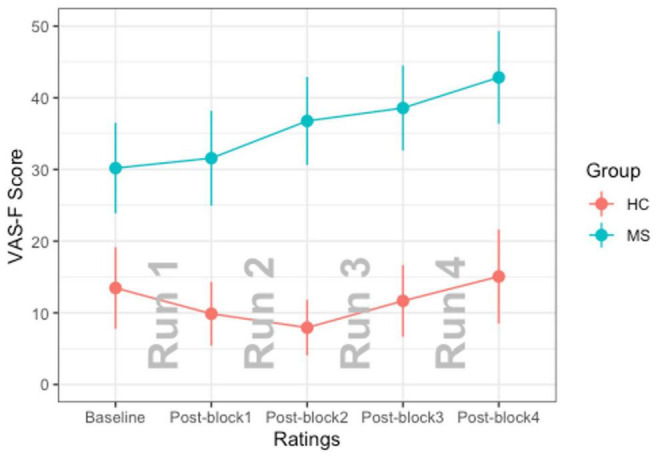
The interaction of Group × Rating is shown. The MS group is plotted in blue and the HC group is plotted in red. Error bars represent standard errors of the mean.

For the analysis of the VAS-F scores, the main effect of Group was significant [*F*(1,38) = 12.97, *p* < 0.001] which was due to individuals with MS reporting higher VAS-F scores than controls (32.5 vs. 9.1, respectively). The main effect of Rating was also significant [*F*(4,156) = 4.39, *p* < 0.01]. This was due to participants reporting increasingly more fatigue across the four runs of each task. The interaction between Group and Rating was also significant [*F*(4,156) = 3.44, *p* < 0.01]. This resulted from the VAS-F scores increasing at a faster rate in the MS group than in the control group (see Table 1 and Figure 1). For all subsequent analyses, we analyzed only data from blocks/runs on which participants reported fatigue scores greater than zero (Wylie et al., 2020). As Table 2 shows, both groups reported experiencing fatigue during well over half of the experimental runs and pwMS reported fatigue on more runs (87%) than did controls (61%) [χ^2^(1) = 14.83, *p* < 0.001].

**TABLE 2:** Number and percentages of runs on which participants reported no fatigue relative to runs where they reported at least some fatigue, as a function of group (Control vs. MS).

	No Fatigue	Fatigue
Control	29 (39%)	46 (61%)
MS	15 (13%)	98 (87%)

### RT and Accuracy

For the RT data, the main effect of Group was significant [*F*(1,34.0) = 3.96, *p* = 0.05], which was due to pwMS responding with longer latencies than the controls (857.8 ms vs. 771.3 ms, respectively). No other effects or interactions were significant.

No significant differences were observed across groups regarding response accuracy, although women showed a trend toward higher accuracy than men [89.9 vs. 83.7%, respectively, *F*(1,32.3) = 3.93, *p* = 0.06]. No other effects or interactions were significant.

### Signal Detection Theory Measures

#### Criterion

The main effect of Group was significant [*F*(1,31.6) = 6.16, *p* < 0.05] and resulted from the MS group responding with a more conservative response bias (0.60) than the control group (0.43). The only other effect to approach conventional levels of significance was VAS-F [*F*(1,58.6) = 3.59, *p* = 0.06], and reflected a positive relationship between VAS-F and response Criterion (coefficient = 0.002). This means that for a unit increase in the VAS-F score, participants’ response bias became more conservative by 0.002. No other effects or interactions were significant.

#### Perceptual Certainty

The only effect to approach conventional levels of significance was that of Sex [*F*(1,38.2) = 3.59, *p* = 0.07]. This resulted from women showing higher Perceptual Certainty (2.40) than men (1.85). No other effects or interactions were significant.

### Structural Neuroimaging Results

We performed three volumetric analyses: we calculated partial correlations between striatal volume and (1) VAS-F, (2) Criterion, and (3) Perceptual Certainty, taking Group membership (control vs. MS) into account. In all cases, the volumetric data was correlated with the fatigue and SDT measures, which were averaged across Task and Run (using only those runs where fatigue was reported). To correct for multiple comparisons, we used the Bonferroni approach, in which family-wise errors are corrected by requiring that the *p*-values are less than 0.05/3 (0.017). The only correlation to approach significance was between striatal volume and Criterion (*r* = −0.34, *p* = 0.026): as striatal volume decreased, participants’ Criterion increased, becoming more conservative. Neither the correlation between striatal volume and VAS-F nor the correlation between striatal volume and Perceptual Certainty approached significance.

### Functional Neuroimaging Results

#### Visual Analogue Scale-F Data

**FIGURE 2:**
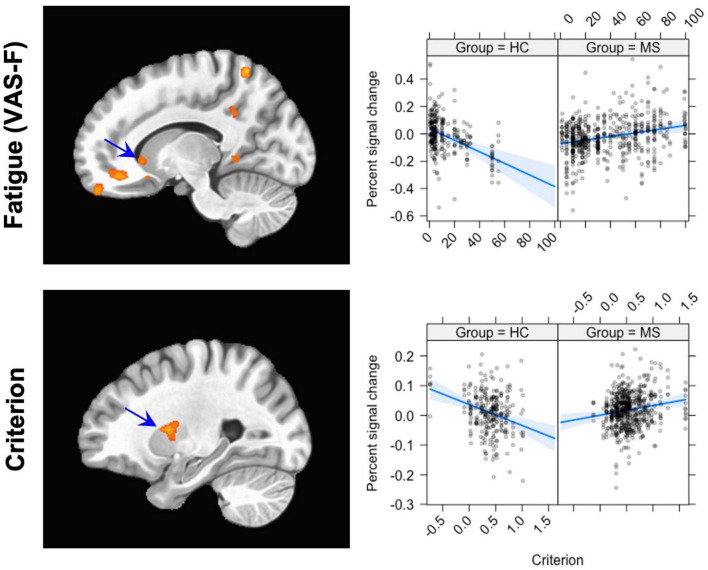
The Group × VAS-F interaction (top row) and the Group x Criterion interaction (bottom row). The panel on the left shows the location of the interaction; the panel on the right shows graphs of the interaction. For the Group × VAS-F interaction, the location plotted is (X, Y, Z = –3, 47, –22); for the Group × Criterion interaction, the location is (X, Y, Z = –27, 2, 10). The scatterplots show the relationship between Percent signal change (ordinate) and VAS-F (abscissa, top) and Criterion (abscissa, bottom). The blue line shows the best fitting linear trend and the blue shaded area shows the 95% confidence interval.

We first investigated the brain activation data associated with cognitive fatigue (VAS-F). There were several areas where brain activation interacted with Group and VAS-F (see Table 3 and Figure 2): vmPFC, the caudate nucleus of the basal ganglia, inferior frontal gyrus, the precuneus, visual areas and cerebellar areas. Figure 2 shows this Group x VAS-F interaction in the caudate nucleus, where the relationship between brain activation and the VAS-F is negative for the control group (coefficient = −0.08) and positive for the MS group (coefficient = 0.04). The same pattern (between brain activation and VAS-F) was seen in the other areas showing a Group x VAS-F interaction in Table 3.

**TABLE 3:** Group × Subjective “State” Fatigue (VAS-F) interactions.

	Fatigue (VAS-F) effects				
Location	X	Y	Z	Voxels	F-stat
**Group**× **VAS-F**					
Superior orbital gyrus/Caudate nucleus	−3.2	46.8	−22	125	25.16
Inferior frontal gyrus	−23.8	19.3	−18	21	16.74
Middle cingulate cortex	−6.6	−42.6	38	17	14.15
Inferior temporal gyrus	58.7	−63.2	−22	35	14.97
Precuneus	−10.1	−56.4	66	19	24.89
Middle occipital gyrus	38.1	−80.4	10	15	11.49
Lingual gyrus	−6.6	−46.1	2	14	13.48
Cerebellum (Crus 1)	27.8	−90.7	−30	47	18.08

*The brain areas associated with the interaction of Group and VAS-F. X, Y, Z the location of the voxel with peak intensity in each cluster; Voxels refers to the number of voxels in the region of overlap. F-stat refers to the F statistic from the voxel with the highest F statistic in the cluster.*

#### Criterion Data

For the Criterion data, there was an interaction between Group and Criterion in several areas, including orbital and middle frontal gyri, the putamen, mid-cingulate cortex, middle temporal gyrus, superior/inferior parietal lobule, the cuneus, and cerebellar regions (see Table 4). Figure 2 shows this interaction in the putamen, and the interaction is qualitatively the same as that seen in the caudate nucleus in the VAS-F data: there was an inverse relationship between Criterion and brain activation for the control group (coefficient = −0.07) and a positive relationship for the MS group (coefficient = 0.03).

**TABLE 4:** Group × Criterion (response bias) interactions.

	Criterion effects				
Location	X	Y	Z	Voxels	F-stat
**Group x Criterion**					
Middle orbital gyrus	55.3	53.7	−10	16	16.98
Middle frontal gyrus	−51.3	57.1	10	17	12.37
Putamen	−27.2	2.1	10	31	18.98
Middle cingulate cortex	10.6	−28.9	26	30	15.64
Middle temporal gyrus	−61.6	−59.8	14	16	14.04
Superior/Inferior parietal lobule	41.5	−63.2	58	16	15.79
Cuneus	−6.6	−83.9	14	39	20.67
Cerebellum(Crus 1)	−37.6	−52.9	−30	16	12.49
Cerebellum(Crus 2)	−20.4	−83.9	−38	17	12.39

*The brain areas associated with the interaction of Group and Criterion. X Y Z = the location of the voxel with peak intensity in each cluster; Voxels refers to the number of voxels in the region of overlap. F-stat refers to the F statistic from the voxel with the highest F statistic in the cluster.*

**FIGURE 3:**
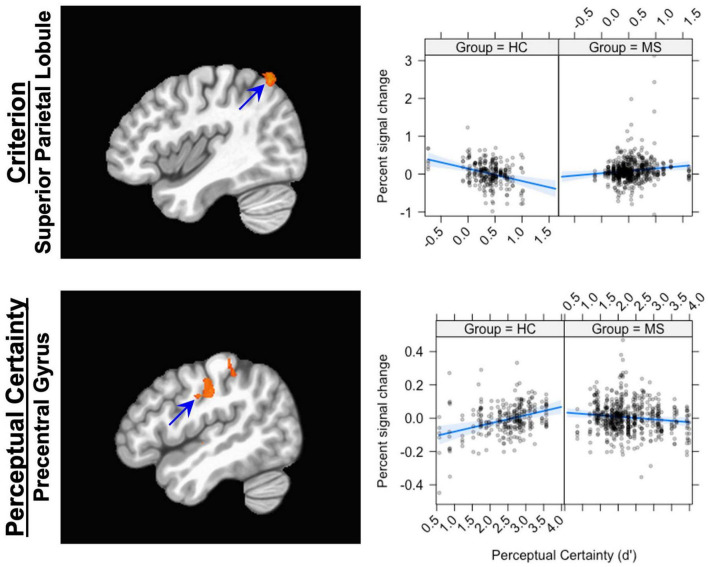
The Group × Criterion interaction (top row) and the Group × d’ interaction (bottom row). The panel on the left shows the location of the interaction; the panel on the left shows graphs of the interaction. For the Group × Criterion interaction, the location plotted is (X, Y, Z = 42, –63, 58); for the Group × d’ interaction, the location is (X, Y, Z = –50, –19, 38). The scatterplots show the relationship between Percent signal change (ordinate) and Criterion (abscissa, top) and Perceptual Certainty (abscissa, bottom). The blue line shows the best fitting linear trend and the blue shaded area shows the 95% confidence interval.

In previous work, we showed that activation in the superior parietal lobule (SPL) covaried with Criterion: increasing activation was associated with decreasing (more liberal) Criterion. The results here show that the relationship between brain activation in the SPL and Criterion was different in the two groups (see Figure 3). Replicating our previous finding, the control group showed a negative relationship between brain activation and Criterion (coefficient = −0.33). The MS group, however, showed a positive relationship (coefficient = 0.13).

#### Perceptual Certainty (d’)

For the Perceptual Certainty data, Group interacted with Perceptual Certainty in the insula, the caudate nucleus, the superior temporal gyrus and the posterior cingulate cortex and several areas around the central sulcus (see Table 5). In previous work, we showed that activation in response-related areas covaried with Perceptual Certainty, with increasing activation associated with increasing Perceptual Certainty. The results here showed the same association in the precentral gyrus (increasing activation associated with increasing Perceptual Certainty), but only for the control group (coefficient = 0.05; see Figure 3). For the MS group, there was an inverse relationship (coefficient = −0.02), such that increasing activation was associated with less Perceptual Certainty.

**TABLE 5:** Group × Perceptual certainty interactions.

	Perceptual Certainty (d’) effects				
Location	X	Y	Z	Voxels	F-stat
**Group × d’**					
Caudate nucleus	−16.9	−15.1	22	15	17.51
Insula	−34.1	−22.0	18	14	14.20
Pre-/Postcentral gyrus	−47.9	−18.5	38	23	13.66
Pre-/Postcentral gyrus	48.4	−25.4	66	37	25.01
Postcentral gyrus	−65.1	−11.7	42	47	18.70
SupraMarginal gyrus	69.0	−18.5	46	27	18.26
Superior temporal gyrus	−44.4	−15.1	−6	16	13.51
Posterior cingulate cortex	0.3	−46.1	6	15	18.90

*The brain areas associated with the interaction of Group and Perceptual Certainty (d’). X Y Z = the location of the voxel with peak intensity in each cluster; Voxels refers to the number of voxels in the region of overlap. F-stat refers to the F statistic from the voxel with the highest F statistic in the cluster.*

### Effects of MS Disease Course

Study results were based on the entire MS sample, including individuals with relapsing and progressive forms of MS (i.e., relapsing-remitting MS, secondary progressive MS, primary progressive MS, and progressive relapsing MS). However, given the potential differences in fatigue and brain functioning in relapsing versus progressive MS subtypes, data analyses were also conducted focusing on the relapsing-remitting MS participants only and yielded substantially the same results, indicating the results were not solely driven by the individuals with more progressive disease courses. The sample size of the progressive MS group (*N* = 6) was too small to conduct separate analyses.

## Discussion

The current study examined SDT metrics – Perceptual Certainty and Criterion – in relation to cognitive fatigue in MS and controls with the primary aim of establishing objective measures of fatigue that align with subjective “state” fatigue ratings. In addition, we determined functional neural correlates of subjective state and objective cognitive fatigue. In line with our first aim, we demonstrated that the SDT metric Criterion (i.e., response bias), was better aligned with state fatigue (VAS-F) than basic performance metrics, such as reaction time and accuracy. When examining the relationship between SDT metrics and subjective “state” fatigue (Aim 2), we found that the MS group became more conservative in their response pattern as their subjective “state” fatigue increased compared to the control group. That is, as fatigue increased, the MS group required more evidence about whether something was a correct or incorrect target before responding during the n-back task blocks. Lastly, we found activation within several brain areas to be associated with subjective state fatigue (VAS-F) and SDT metrics in both the MS and control groups (Aim 3), though the direction of these relationships differed by group, such that state fatigue (VAS-F) and Criterion (i.e., response bias) showed a positive correlation with brain activation, while Perceptual Certainty showed an inverse correlation with brain activation in the MS group. The opposite was true for the control group (i.e., inverse correlations between state fatigue and response bias and brain activation, positive correlation between Perceptual Certainty and brain activation).

One important finding of the current study was our demonstration that the SDT metric Criterion (i.e., response bias), a proposed objective measure of cognitive fatigue, was related to subjective state fatigue in MS. These results build upon our previous work that showed the same relationship in a group of controls (Wylie et al., 2020), thereby laying the foundation for a viable metric of objective cognitive fatigue. The finding of a Criterion-subjective fatigue relationship in MS is further strengthened by the redemonstration of previous study findings (Craig and Cooper, 1992; Stoner, 1996; Torres-Harding and Jason, 2005) showing that performance measures such as reaction time and accuracy are poorly correlated with subjective fatigue ratings. What traditional performance measures fail to capture is the tradeoff between effort and reward (i.e., the payoff matrix) which has been shown to be highly relevant for cognitive fatigue (Dobryakova et al., 2013, 2015; Wylie et al. 2017b, 2020) and *is* captured by SDT metrics, especially Criterion. We show that at high levels of fatigue the MS group’s *approach* to performance changes (i.e., the MS group adopts a more conservative response bias) due to effort increasing while reward remains unchanged, and it is our hypothesis that this high effort low reward tradeoff is experienced as fatigue. Thus, fatigue should be reduced if subjects are given a reward for performing a fatiguing task – a prediction that we have verified in both MS (Dobryakova et al., 2018) and in individuals who have sustained a TBI (Dobryakova et al., 2020). Future work will explore how the introduction of additional reward after the induction of fatigue moderates the relationship between subjective state fatigue and SDT metrics.

Another notable result was the connection between SDT variables and brain activation which establishes a link between the brain and objective measures of fatigue in MS. Previous work has implicated the basal ganglia, particularly the caudate, in subjective cognitive fatigue (Chaudhuri and Behan, 2004; Dobryakova et al., 2013, 2017; Nakagawa et al., 2016) and to a lesser degree more objective measures of cognitive fatigue (in controls; Wylie et al., 2020). Here, we demonstrated relationships between subjective state fatigue (i.e., VAS-F) and response bias (i.e., Criterion) and activation in the basal ganglia, as well as several other regions throughout the brain, which is consistent with previous research identifying a functional “fatigue network” (Wylie et al., 2020). The network identified by Wylie et al. (2020) included the striatum of the basal ganglia, dorsolateral prefrontal cortex, dorsal anterior cingulate cortex, ventromedial prefrontal cortex, anterior insula, and additional frontal regions. A separate study identified similar areas in fatigue processing, adding that several of these areas are also implicated in reward processing (Chen et al., 2020). Thus, given the overlap in activation in similar brain areas in the current study, it is plausible that SDT variables, namely Criterion, capture fatigue and reward processing networks. This has implications for our understanding of cognitive fatigue and how to properly quantify it. Regarding activation, contrary to the controls, the MS group showed positive relationships (i.e., as Criterion increased, brain activation increased). This positive relationship may be driven by the increased effort required by more neurologically compromised brains (i.e., “hyperconnectivity”) to complete tasks, especially in the context of a fixed reward, thereby keeping this network engaged for longer periods of time and resulting in the perception of fatigue. This hyperconnectivity has also been demonstrated in other studies across diverse neurological populations (Hillary et al., 2015; Jie et al., 2016; Pravatà et al., 2016; Rosskopf et al., 2018). Lastly, results from structural MRI showed an inverse relationship between the striatum and response bias (i.e., Criterion), such that less striatal volume was associated with more conservative response bias. Though these results were only significant at a trend level, they are promising, because they replicate previous anatomical patterns of striatal atrophy in relation to fatigue in MS and other neurological populations and controls (Calabrese et al., 2010; Damasceno et al., 2016; Nakagawa et al., 2016; Kluger et al., 2019; Palotai et al., 2020; Wylie et al., 2020; Schnellbächer et al., 2021). Our results thus further underline the importance of the striatum in the experience of cognitive fatigue.

Basic performance measures (i.e., reaction time, accuracy) and questionnaires aimed at quantifying cognitive fatigue have fallen short, hindering our ability to accurately appraise fatigue and develop interventions. Thus, the current study begins to fill a gap in the scientific literature by linking patients’ experience of fatigue with objective measures in a way that has not been done before and illuminating the connection between pathophysiology and novel measures of cognitive fatigue by way of SDT. These results hold promise for characterizing cognitive fatigue in MS and other neurological populations in the future.

## Limitations and Future Directions

While our results largely supported our proposed hypotheses, there are still notable limitations. First, participants were not offered rewards over time in this study, which limited our ability to test whether Criterion (i.e., response bias) changes in relation to varying levels of reward. Integrating the distribution of rewards in future studies will be crucial for testing whether the payoff matrix is playing a role in cognitive fatigue. Our sample size was also relatively small, and we did not have access to crucial sociocultural demographic variables (e.g., race/ethnicity) that may have impacted the perception of fatigue. It has been well-established that sociocultural factors influence cognitive performance and perceptions of disease-associated symptoms, so recruiting more diverse populations and integrating cultural factors in the future will be crucial for understanding cognitive fatigue and how our findings generalize to a more representative MS population. The lack of assessment of additional clinical factors that might lead to cognitive fatigue, such as affective symptoms, sleep quality, or current use of disease modifying treatments, was also lacking in our investigation. Future studies would benefit from using multilevel modeling to investigate how multiple variables contribute to cognitive fatigue. Our sample was largely made up of participants with RRMS which has a different underlying mechanism than progressive courses of MS. Thus, it will be important to replicate these findings in a sample of individuals with progressive MS subtypes. Lastly, our analyses of brain metrics were restricted to activation of brain areas on an individual scale, rather than through network analyses. The brain is not modular, so the examination of brain networks is crucial for augmenting our understanding of the neurophysiological processes that contribute to and moderate fatigue. Thus, future works could benefit from examining the relationship between SDT variables and structural and functional brain networks, particularly those thought to be associated with fatigue and reward processes. Incorporating physiological measures such as event-related or evoked potentials would also help to further unravel the underlying mechanisms of cognitive fatigue in MS.

## Conclusion

Our results demonstrate the relationship between subjective “state” fatigue and response bias (i.e., Criterion). In addition, not only do we show that subjective state fatigue is related to brain activation in areas associated with a previously demonstrated functional “fatigue network,” but brain activation in these same regions is also related to response bias (i.e., Criterion). Therefore, accounting for more nuanced and complex aspects of performance (as captured by SDT Criterion) brings us much closer to understanding the pathophysiology of cognitive fatigue compared to traditional performance measures. These results hold promise for characterizing cognitive fatigue in MS and developing effective interventions in the future.

## Data Availability Statement

The raw data supporting the conclusions of this article will be made available by the authors, without undue reservation.

## Ethics Statement

The studies involving human participants were reviewed and approved by Kessler Foundation Institutional Review Board. The patients/participants provided their written informed consent to participate in this study.

## Author Contributions

CR and GW wrote the main manuscript. JD, BY, and HG reviewed and edited the manuscript. All authors contributed to the article and approved the submitted version.

## Conflict of Interest

The authors declare that the research was conducted in the absence of any commercial or financial relationships that could be construed as a potential conflict of interest.

## Publisher’s Note

All claims expressed in this article are solely those of the authors and do not necessarily represent those of their affiliated organizations, or those of the publisher, the editors and the reviewers. Any product that may be evaluated in this article, or claim that may be made by its manufacturer, is not guaranteed or endorsed by the publisher.

## Footnotes

1
https://surfer.nmr.mgh.harvard.edu/optseq/
